# Detection of Germanium Nanocrystals as Tracer Materials in Polypropylene via Raman Spectroscopy

**DOI:** 10.3390/ma19112185

**Published:** 2026-05-22

**Authors:** Monique Greiner, Michael Pohlitz, Philipp Kitschke, Aylin Last, Christian K. Müller, Jonathan G. C. Veinot, Michael Heinrich

**Affiliations:** 1 Institute for Production Technology, Faculty of Automotive and Mechanical Engineering, University of Applied Sciences Zwickau, 08056 Zwickau, Germany; 2 Leupold Institute of Applied Sciences, Faculty of Physical Engineering/Computer Sciences, University of Applied Sciences Zwickau, 08056 Zwickau, Germany; michael.pohlitz@whz.de (M.P.); philipp.kitschke@whz.de (P.K.); aylin.last@whz.de (A.L.); christian.mueller.1@whz.de (C.K.M.); 3 Department of Chemistry, University of Alberta, Edmonton, AB T6G 2G2, Canada; jveinot@ualberta.ca

**Keywords:** germanium nanocrystals, polypropylene, composites, Raman spectroscopy, tracer technology, digital material passport

## Abstract

**Highlights:**

- Utilization of germanium nanocrystals (GeNCs) as tracer materials for polymers.
- Synthesis and functionalization with dodecyl ligands of hydride-terminated GeNCs.
- Integration of GeNCs into polypropylene in different concentrations.
- Spatially resolved Raman mapping of GeNCs in polypropylene.

**Abstract:**

Increasing regulatory demands for high-quality plastic recycling create a strong need for novel tracer systems that enable reliable polymer identification and sorting. This feasibility study evaluates germanium nanocrystals (GeNCs) as Raman-detectable tracer materials in polypropylene (PP). The synthesis of GeNC/PP composite materials possessing various GeNC contents via a solvent-based intercalation process followed by compounding and injection molding is reported. Hydride-terminated GeNCs were synthesized and subsequently functionalized with dodecyl ligands to ensure chemical stability, compatibility with the polymer matrix, and processability under conventional melt-processing conditions. The dodecyl-functionalized GeNCs were successfully stabilized and homogeneously integrated into the PP matrix. Raman spectroscopy demonstrates the clear detection of GeNCs within the composites through a characteristic Ge–Ge optical phonon mode at 296 cm^−1^, which is well separated from the intrinsic Raman bands of polypropylene. The Raman signal intensity increases systematically with increasing GeNC concentration. Raman mapping reveals an overall homogeneous distribution of the nanocrystals within the polymer, while a slight tendency toward agglomeration is observed at higher loadings. These results demonstrate that GeNCs are well suited as optically detectable tracers for polypropylene and can be reliably identified using Raman spectroscopy, highlighting their potential for tracer-based sorting concepts in advanced recycling and digital material passport applications.

## 1. Introduction

Recent European Union regulations, including the Packaging and Packaging Waste Regulation (PPWR) and the Circular Economy Action Plan, mandate substantial increases in recycling rates and the use of recycled content in new products [1,2]. The volume of plastic waste is projected to double by 2050 [3], underscoring the urgent need to enhance recycling rates. Currently, just under 50% of post-consumer plastics waste in Europe undergoes thermal utilization rather than mechanical or chemical recycling [4]. A significant factor contributing to these low recycling rates are the inherent limitations of conventional sorting processes, which predominantly rely on near-infrared (NIR) spectroscopy. Black plastics, which are frequently used in technical applications, cannot be identified to any great extent using NIR [5]. High-quality recyclates necessitate well-sorted waste streams that account not only for polymer type but also for the presence of additives, fillers, and reinforcement materials such as fibers [6]. The accurate identification of these components typically requires the integration of multiple analytical technologies. To overcome the limitations of conventional sorting facilities alternative methods such as Fourier transform infrared spectroscopy (FTIR), Raman-spectroscopy, laser-induced breakdown spectroscopy (LIBS), X-ray fluorescence spectroscopy (XRFS), hyperspectral imaging, terahertz imaging, and machine learning were investigated to improve mentioned detection systems [7].

Another promising approach for improved sorting is tracer-based sorting (TBS), whereby the detection system does not identify the plastic itself by its characteristics but rather a tracer material with specific optical features. The idea to use photoluminescent tracers for plastics identification and sorting first occurred in a patent in 1991 [8]. Since then, suitable marking materials and analysis or sorting methods as well as various options for integrating or applying the tracer materials in or on the plastic products have been investigated. These tracer materials include, among others, rare earth oxides (lanthanides), Carbon Quantum Dots and supraparticles with a magnetic fingerprint, as well as intrinsically luminescent polymer-based materials. However, tracer material-specific disadvantages such as toxicity and poor photostability were identified [9,10,11,12].

The TBS concept allows creating an optical fingerprint that acts as a material passport, encoding information about polymer types, regardless of color, as well as details regarding additives, fillers and other constituents. Utilizing such advances may help to reduce greenhouse gas emissions by enabling higher mechanical and chemical recycling rates as alternatives to thermal recovery. Moreover, cross-contamination with hazardous substances, such as flame retardants, throughout the recycling process can be omitted. As a result, highly refined waste streams yielding superior-quality recyclates suitable for high-value applications, including electronics housings and automotive components, are available and thereby reducing the dependence on virgin materials.

While the TBS concept demonstrates considerable potential, the practical implementation requires tracer materials that combine excellent optical properties with non-toxicity, photostability, and processability. Among the emerging alternatives, semiconductor nanocrystals (NCs) represent an attractive option in this regard. These materials have received significant interest due to their tunable properties making them attractive for sophisticated modern applications such as bio-imaging, sensors, lasers, light emitting diodes, memory elements, and for energy conversions [13,14,15,16]. Their potential applications are wide-ranging, including use as materials for nanostructured light sources, sensors, field-effect transistors, solar cells, security technologies, imaging processes and drug delivery in biological systems [13,14,15,16,17,18,19,20,21,22,23,24]. However, the most widely studied NCs are based upon toxic elements such as Pb, Cd or Hg [25], which limits the practical utility based upon these systems insofar as their acceptance as materials for everyday applications. In contrast, Group 14 semiconductors (i.e., Si and Ge) are abundant, possess the same size-tunable properties of other NCs, and reports indicate that nanomaterials based upon these elements are non-toxic [13,14,21,26]. Intensive research into the targeted surface functionalization of Group 14 nanocrystals, particularly silicon nanocrystals (SiNCs), has been conducted over the past few decades [27,28,29,30,31,32,33,34,35,36,37,38,39,40]. Recently, studies based on this research have been published, presenting the use of functionalized SiNCs in modern applications such as product security (anti-counterfeiting) and photodynamic cancer therapy [41,42]. Moreover, composite materials consisting of SiNCs and polymeric materials can be produced without any segregation effects occurring. This allows the unique optoelectronic features of the SiNCs to be combined with the properties of the polymer matrix [43,44,45]. In contrast, studies focusing on the practical use of GeNCs are rarely reported, with most research centering on their application in the modern semiconductor industry, for instance in solar cells [46,47] and quantum computing [48,49]. To the best of our knowledge, the incorporation of GeNCs into an organic polymer matrix has only been reported once, by Uyar and coworkers. In this study, an electrospinning technique was used to give a composite material based on GeNCs and polyvinyl alcohol nanofibers (GeNC/PVA) that combines the optoelectrical properties of the GeNCs with the features of the PVA matrix [50]. The latter as well as the studies reporting on the manufacturing of SiNCs/polymer composite materials using tailor-made surface-functionalized SiNCs motivated us to investigate whether this general concept could be applied using surface-functionalized GeNCs for marking and identifying bulk plastics for their recyclable use.

Here, we report on the synthesis of composite materials via a solvent-based intercalation process using dodecyl-functionalized GeNCs and commercially available polypropylene (PP) granules. Using a masterbatch approach, PP composites with different GeNC contents are compounded and further processed into test specimens using injection molding technology. Based on the obtained GeNC/PP composites, we demonstrate that dodecyl-functionalized GeNCs can be used to mark plastics and facilitate their identification during the recycling process using Raman spectroscopy as a reliable analysis method. Raman spectroscopy was found to be a suitable analytical method because spectroscopic fingerprints of GeNCs can be easily distinguished from the characteristic vibrations of polymers. These spectroscopic fingerprints can be adjusted with respect to wavenumber by varying the size of the NCs, allowing the use of defined NCs for specific polymers. In a further step, the adjustable spectral fingerprints allow us to obtain spectral material passports that enable the identification of the type of polymer as well as other compounds such as additives or fillers. However, the present study focuses on the integration and detection of GeNCs in polypropylene via Raman spectroscopy as a step towards the development of NC-based material passports. To the best of our knowledge, this work represents the first successful demonstration of this concept in GeNC/bulk polymer composites, opening new opportunities for advanced material identification and traceability.

## 2. Materials and Methods

### 2.1. Materials

Germanium dioxide powder (GeO_2_, 99.9%) was purchased from ChemPur Feinchemikalien und Forschungsbedarf GmbH (Karlsruhe, Germany). Hypophosphorous acid (50 wt% in H_2_O) and 1-dodecene (97%) were purchased from Thermo Fisher GmbH (Darmstadt, Germany). Sodium hydroxide pellets, reagent grade methanol, toluene, mesitylene, ethanol, *n*-hexane, hydrofluoric acid (HF, 49% aqueous solution), hydrochloric acid (37%), ammonium hydroxide solution (28–30%) and Polytetrafluorethylene (PTFE) syringe filters ($d_{pore}=\;0.45\;\mu m$) were purchased from VWR International GmbH (Darmstadt, Germany). A virgin polypropylene homopolymer (Sabic^®^ PP 571P) was purchased from PiO Kunststoffe GmbH (Freiburg im Breisgau, Germany). All reactions were performed under argon using standard Schlenk techniques or in a glovebox. Solvents were purified and dried by applying standard techniques. The reactions were carried out with solvents that were freshly distilled from appropriate drying reagents immediately prior usage. Unless otherwise indicated, reagents were used as received.

Hydride-terminated germanium nanocrystals (H-GeNCs) were obtained starting from GeO_2_ powder according to a synthesis protocol reported by Veinot and coworkers previously [51]. Please note that the synthesis of the Ge@GeO_2_ hybrid material and the consecutively formed H-GeNCs were performed in the absence of silicon grease avoiding any source of silicon atom contamination for the final products. The H-GeNCs were always freshly liberated from Ge@GeO_2_ hybrid materials (about 100 mg) by HF etching and freed from residual solvents under vacuum ($p\;\sim \;10^{-2}\;mbar$) for at least one hour after work-up procedure and before usage. Starting from m ~ 100 mg of Ge@GeO_2_ hybrid material yielded on average m ~ 31 mg of H-GeNCs as brown powder.

### 2.2. Material Characterization and Instrumentation

Fourier transform infrared spectroscopy was performed using a Nicolet Magna 750 IR spectrophotometer (Thermo Fisher Scientific, Darmstadt, Germany). Samples were drop-cast from suspensions containing the material in question using either *n*-hexane or toluene as liquid phase. X-ray powder diffraction (PXRD) was carried out using an INEL XRG 3000 X-ray diffractometer (Thermo Fisher Scientific, Darmstadt, Germany) equipped with a Cu-K*α* radiation source (λ=1.54 Å) and CPS-120 detector. The crystallite size was estimated using the Scherrer equation: $\tau =\frac{K\lambda }{\beta cos\theta }$; here *τ* is the volume-weighted crystallite size, *K* is the Scherrer constant here taken as 0.94, *λ* is the X-ray wavelength, θ is the Bragg angle in ° and *β* is the full width of the diffraction line at half of the maximum intensity (FWHM, background subtracted). Bright-field transmission electron microscopy (TEM) and energy dispersive X-ray (EDX) analyses were performed using a JEOL-2010 (LaB_6_ filament) electron microscope (JEOL, Freising, Germany) with an accelerating voltage of 200 kV. TEM samples of GeNCs were prepared by drop-coating of 1–3 drops of a dilute dispersion containing the material of interest using either *n*-hexane or toluene as liquid phase onto a holey carbon coated copper grid (300 mesh, Electron Microscopy Science, Morgantown, PA, USA). Bright-field TEM images were processed using ImageJ software (version 1.51s). Particle size distributions were determined by measuring at least 500 nanocrystals. Raman spectroscopy for the analysis of the functionalized GeNCs was carried out using a Renishaw inVia Raman microscope (Renishaw, Gloucestershire, United Kingdom) equipped with a 514 nm diode laser and a power of 3.98 mW on the sample. Samples were prepared by mounting the dispersions on either gold-coated glass, copper foil or silicon wafers. Raman measurements of the GeNC/PP composites were carried out using an Oxford RISE confocal Raman microscope (Oxford Instruments, Abingdon, United Kingdom). Excitation was provided by a 532 nm laser operated at 0.6 mW to avoid photothermal effects in the polypropylene matrix. Single-point spectra were collected with an integration time of 0.7 s and 100 accumulations. Raman mapping was performed over a $30\;\times \;30\;{µm}^{2}$ region using 80 × 80 pixels, with an integration time of 0.6 s per pixel. Raw spectra were pre-processed by cosmic-ray removal and smoothed using the instrument’s built-in filter. X-ray photoelectron spectra were acquired in energy spectrum mode at 210 W, using a Kratos Axis Ultra X-ray photoelectron spectrometer (Kratos Analytical Ltd., Manchester, United Kingdom). X-ray source was Al (Mono) K*α* line with an energy of 1486.6 eV. The probing area was about $2\;{mm}^{2}$. Samples were prepared as films by drop-casting suspensions of the material in question in either *n*-hexane or toluene onto copper foil substrates. Binding energies were calibrated using the C 1 s peak as a reference (284.8 eV). CasaXPS Version 2.3.18PR1.0 software was used for X-ray photoelectron spectroscopy (XPS) data analyses. Peak fitting was performed after background subtraction (Shirley type). The high-resolution Ge 3d region of the XP spectra were collected for all samples investigated and were fitted to Ge 3d_3/2_/Ge 3d_5/2_ partner lines with spin–orbit splitting fixed at 0.58 eV, and the Ge 3d_3/2_/Ge 3d_5/2_ intensity ratio was set to 0.67.

Graphical images of analytical data were created using the Origin^®^ Pro program package (version 2022) [52] and chemical structures were depicted using the ChemDraw 12 [53] program package.

### 2.3. Synthesis of Dodecane-Terminated GeNCs

H-GeNCs (~31 mg) were dispersed in 1-dodecene (~10 mL) under argon and three freeze–pump–thaw cycles [a cycle is defined as follows: freezing the dispersion using liquid nitrogen until the liquid became a solid; then applying vacuum ($p\;\sim \;10^{-2}\;mbar$) for at least 15 min followed by heating the mixture to ambient temperature] were performed. The cloudy, oxygen-free reaction mixture was then heated to 190 °C overnight with stirring. The dodecane-terminated GeNCs were isolated and purified using a solvent/antisolvent precipitation procedure. The crude reaction mixture was transferred to polypropylene centrifuge tubes and about 45 mL of an ethanol/methanol (v/v ~ 1/1) mixture acting as an antisolvent was added. The resulting cloudy brown suspension was centrifuged at 6000 rpm for 10 min to yield a brown precipitate and a transparent supernatant. The supernatant was discarded by decantation. The brown precipitate was redispersed by ultrasonic treatment in a minimum volume of toluene (~2 mL). Subsequently, methanol (~50 mL) was added to yield a cloudy suspension that was centrifuged at 6000 rpm for 10 min to yield a brown precipitate and a clear supernatant. The supernatant was discarded by decantation. The latter suspension/centrifugation procedure was repeated one more time. The resulting precipitate was then redispersed by ultrasonic treatment either in *n*-hexane (for further analysis) or in mesitylene (for further use in synthesis). Yield (on average): m=61 mg.

Further treatment of the dodecyl-functionalized GeNCs dispersed in *n*-hexane prior to analyses: The dispersion was allowed to age for at least 15 h resulting in sedimentation of non-stable particles. The cloudy supernatant was isolated using a syringe and then filtered using a PTFE syringe filter ($d_{pore}=0.45\;\mu m$) to give a stable dispersion of surface-functionalized GeNCs in *n*-hexane to be used for analyses. Please note that a glass syringe was used instead of any other plastic syringes to avoid contamination of the final product by chemicals such as softeners.

### 2.4. Synthesis of the GeNC/PP Composite (Masterbatch Preparation)

A homogeneous suspension of dodecyl-functionalized GeNCs (~54 mg) in mesitylene (~1 mL) was added to a solution of polypropylene (~470 mg) in mesitylene (~7.5 mL) at 130 °C, with stirring of the reaction mixture throughout. Removal of all volatiles under reduced pressure (3 mbar) at 75 °C yielded GeNC/PP composite material as a gray powder. For Raman measurements ~82 mg were extracted which leads to a yield of: m=442 mg.

### 2.5. Manufacturing of GeNC/PP Test Specimens

Prior to processing, the polymer materials were dried at 100 °C for 24 h. Four batches with varying GeNC concentrations were prepared by diluting a masterbatch containing 10.31 wt% GeNCs with virgin polypropylene (PP). The total weight of each batch was maintained at approximately 6150 mg, which corresponds to the processing capacity of the compounder utilized.

Table 1 summarizes the composition and target tracer concentrations for each batch. Thermal stability of the NCs is given as polymer processing is carried out at temperatures ≤190 °C, which is the functionalization temperature of the GeNCs.

Compounding was performed using a Thermo Scientific HAAKE MiniLab 3 twin-screw extruder (Thermo Electron, Karlsruhe, Germany) equipped with co-rotating screws. Each batch was processed at a barrel temperature of 190 °C and a screw speed of 100 rpm. The material was recirculated over an integrated backflow channel and a bypass valve within the compounder for 150 s to ensure homogeneous dispersion of the GeNCs throughout the polymer matrix.

**Figure 1:**
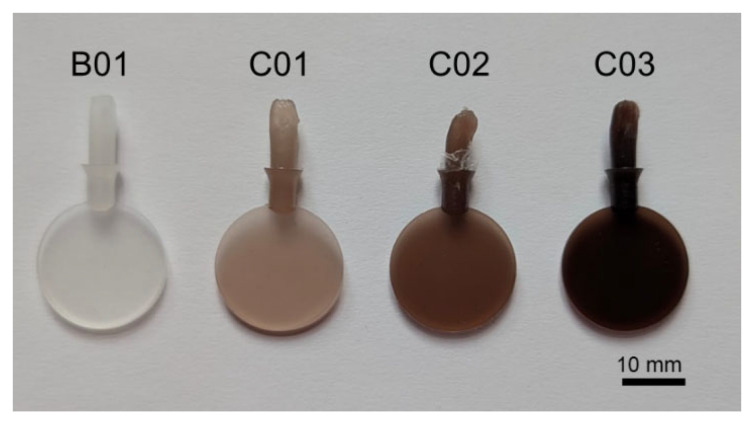
Injection molded specimens containing 0 wt% (B01), 0.06 wt% (C01), 0.23 wt% (C02) and 0.46 wt% (C03) GeNCs.

The compounded polymer melt was subsequently extruded directly into the heated cylinder of a Thermo Scientific HAAKE MiniJet Pro injection molding machine (Thermo Electron, Karlsruhe, Germany). Disk-shaped specimens with a diameter of 20 mm and a thickness of 1.5 mm were fabricated via injection molding. The cylinder temperature was maintained at 190 °C, while the mold temperature was set to 50 °C. An injection pressure of 100 bar was applied for 5 s, followed by a holding pressure of 150 bar. The Injection molded specimens containing 0 wt% (B01), 0.06 wt% (C01), 0.23 wt% (C02) and 0.46 wt% (C03) GeNCs in Figure 1 appear opaquer with increasing GeNC content. On a macroscopic scale the distribution of the GeNCs seems homogeneous.

## 3. Results and Discussion

### 3.1. Synthesis and Characterization of GeNCs

**Scheme 1:**
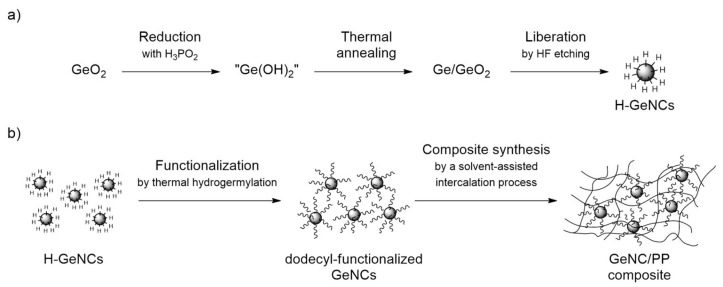
Synthesis of (**a**) H-GeNCs starting from GeO_2_ according to a procedure reported by Veinot and coworkers [40] and (**b**) preparation of the GeNCs/PP composite using dodecyl-functionalized GeNCs, which were obtained by a thermal hydrogermylation reaction of H-GeNCs.

Dodecyl-functionalized GeNCs were synthesized starting from freshly prepared hydride-terminated GeNCs (H-GeNCs) using a thermal hydrogermylation reaction according to synthesis protocols reported by Veinot and coworkers [40]. A brief outline of a combined protocol is given as follows. Reduction in GeO_2_ yielded “Ge(OH)_2_”, which was annealed at 400 °C for an hour to form a Ge@GeO_2_ hybrid material containing GeNCs. Liberation of the GeNCs was performed by an etching process applying HF resulting in H-GeNCs (Scheme 1a). Freshly prepared H-GeNCs were always used for further functionalization reactions. Solvent-freed H-GeNCs were suspended in 1-dedecene and heated to 190 °C overnight to carry out the thermal hydrogermylation reaction for the alkyl-functionalization of the GeNCs. The dodecyl-functionalized GeNCs were isolated by a solvent/antisolvent precipitation procedure. They were then used, without any further chemical modification, to prepare the GeNCs/PP composite via a solvent-assisted intercalation process (Scheme 1b).

**Figure 2:**
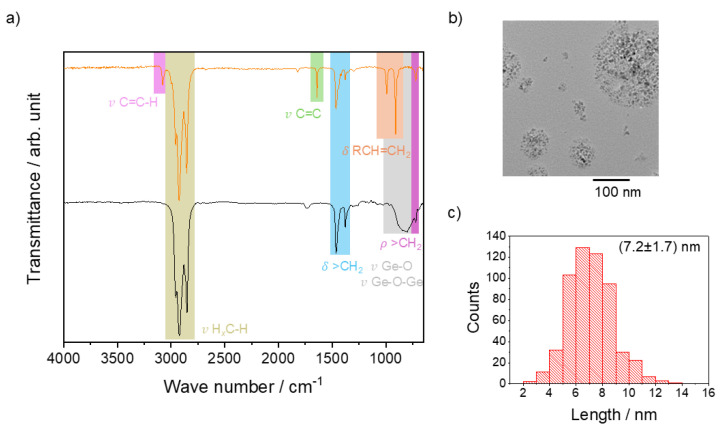
(**a**) FTIR spectra of dodecyl-functionalized GeNCs (black line) and 1-dodecane (orange line) as a reference are depicted. Absorption bands being indicative of specific vibrational modes are highlighted. (**b**) A representative TEM image of the dodecyl-functionalized GeNCs and the corresponding (**c**) size distribution diagram illustrating the average particle size determined by counting at least 500 particles are shown.

The dodecyl-functionalized GeNCs were characterized using FTIR spectroscopy, TEM, XRD, XPS and Raman spectroscopy (Figure 2 and Figures S1–S3). All analytical data confirm the formation of alkyl-functionalized GeNCs and are in agreement with the values reported in the literature [40,47,50,54]. FTIR spectroscopy analysis indicates that the hydride surface functions (H-Ge) of the H-GeNCs (intermediates) have been fully converted into dodecyl groups by the hydrogermylation reaction. The absorption band centered at $\bar{v}=820\;{cm}^{-1}$ is assigned to residual germanium oxide species resulting from incomplete etching during the liberation process (Figure 2a). The latter is supported by XRD data. Reflections of low intensity that are attributed to GeO_2_ were detected for the dodecyl-functionalized GeNCs (Figure S1). A representative bright-field transmission electron microscopy (TEM) image of the dodecyl-terminated GeNCs is shown in Figure 2b. The particles appear pseudospherical with average diameters (determined by counting at least 500 particles) slightly larger than 7 nm (Figure 2c) agreeing with XRD data (Figure S1).

### 3.2. Characterization of GeNC-Doped Specimen

**Figure 3:**
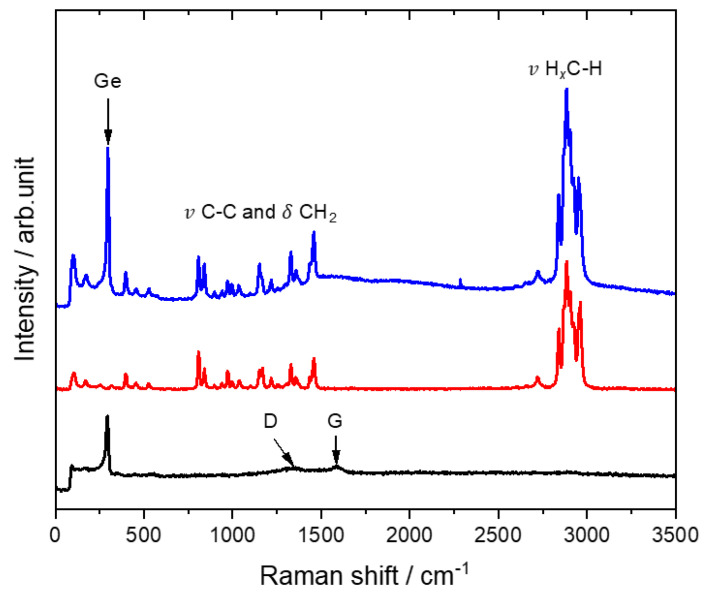
Raman spectra of dodecyl-functionalized GeNCs (black), polypropylene (red), and GeNC/PP composite material (blue). The composite spectrum exhibits characteristic vibrational signatures of both components.

Raman spectra of PP, GeNCs and GeNC/PP composites are summarized in Figure 3. Polypropylene exhibits a well-characterized Raman spectrum with distinct bands in both the fingerprint region (below $1500\;{cm}^{-1}$) and the high-frequency C–H stretching region (2800–3000 ${cm}^{-1}$) [55,56].

In the case of the functionalized GeNCs (Figure 3, black graph), a strong peak appears at $\sim 296\;{cm}^{-1}$, which corresponds to the Ge–Ge optical phonon mode, confirming the presence of crystalline germanium nanoparticles. In addition, two broad carbon-related bands are observed at approximately $1330\;{cm}^{-1}$ and $1590\;{cm}^{-1}$, corresponding to the D and G bands of graphitic carbon.

The Raman spectrum of the GeNC/PP composite (Figure 3, blue graph) combines the characteristic signatures of both constituents. The typical polypropylene bands remain visible in the fingerprint region between ~800–1500 ${cm}^{-1}$, associated with C–C stretching vibrations and CH_2_/CH_3_ deformation modes, and between ~2700–3000 ${cm}^{-1}$ due to distinct C–H stretching bands, while the Ge-related Raman band at $296\;{cm}^{-1}$ is additionally present (Figure 3). In contrast, pure PP (Figure 3, red graph) exhibits no Raman features in this spectral region.

**Figure 4:**
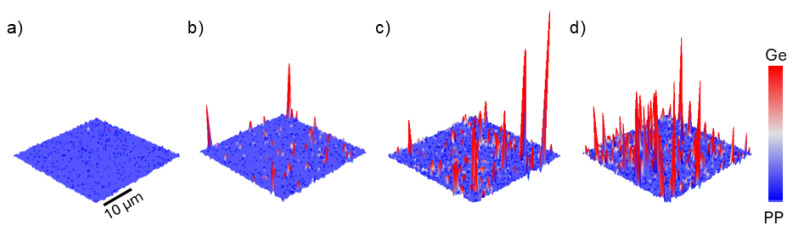
Raman intensity maps of the $\sim 296\;{cm}^{-1}$ Ge–Ge phonon mode for (**a**) pure PP (B01) and polypropylene composites with increasing GeNC loading: (**b**) C01, (**c**) C02, and (**d**) C03. The intensity scale (blue to red) corresponds to the charge coupled device (CCD) signal amplitude at the characteristic Ge Raman band. High-intensity regions in the generated images (displayed in red) correspond to locations with enhanced Ge Raman signal, indicating localized nanoparticle presence or increased scattering efficiency. All maps cover a $30\;\times \;30\;{µm}^{2}$ area.

To investigate the nanoparticle distribution, Raman intensity maps of the $\sim 296\;{cm}^{-1}$ Ge–Ge band were recorded over an area of $30\times 30\;{µm}^{2}$ (80 × 80 pixels) for all samples (Figure 4). These false-color maps vividly illustrate how the Ge nanoparticles are distributed within the PP matrix. The pure PP sample B01 showed no detectable signal at $296\;{cm}^{-1}$ and only a homogeneous background with minor noise fluctuations (Figure 4a). In sample C01, containing the lowest GeNC concentration, isolated low-intensity spots appeared above the background (Figure 4b). These signals were spatially separated and sparsely distributed. For sample C02, the number and intensity of the Ge-related spots increased markedly (Figure 4c). The GeNC signals were more evenly distributed across the mapped area, although localized regions with several neighboring particles became visible. Finally, the highest loading sample (C03) exhibited a dense array of intense signals in the $296\;{cm}^{-1}$ map (Figure 4d). Several neighboring signals merged into larger regions of elevated intensity, indicating the formation of extended Ge-rich domains.

Elemental germanium has a well-known first-order Raman-active mode in the vicinity of $300\;{cm}^{-1}$. In the diamond-cubic crystal structure of Ge (often referred to as the Ge-I phase), the fundamental optical phonon gives rise to a Raman signal at approximately $300\;{cm}^{-1}$, corresponding to the Ge–Ge bond vibrational mode. For example, bulk crystalline Ge typically shows a sharp Raman band at $300\;{cm}^{-1}$, which is the longitudinal optical phonon mode of the Ge lattice [57]. When germanium is present as nanocrystals, the Raman features remain centered around the same general region but can exhibit slight shifts and broadening due to phonon confinement and strain effects. Notably, the principal Raman line for Ge nanoparticles is often reported near $\sim 297\;{cm}^{-1}$, a few ${cm}^{-1}$ lower than the bulk value. This shift to $\sim 296\;{cm}^{-1}$ (and generally a broader peak) is a signature of nanocrystalline Ge. Indeed, one study of laser-synthesized GeNCs observed the Ge–Ge mode at about $293-297\;{cm}^{-1}$ for the nanocrystals, appearing as an asymmetric broadened peak [58,59].

The prominent D ($\sim 1330\;{cm}^{-1}$) and G ($\sim 1590\;{cm}^{-1}$) C-bands appearing in the GeNC spectrum (Figure 3, black graph) indicate the presence of graphitic carbon species generated from the organic ligands. The emergence of these bands has been observed upon ligand breakdown, correlating with carbonaceous residue formation on the nanocrystals [60]. Such D/G features have been reported in similar systems after the decomposition of dodecyl ligands [61,62].

The GeNC/PP composite spectra demonstrate that the Ge-related Raman signal can be clearly distinguished from the polypropylene matrix (Figure 4). Since pure PP does not exhibit any Raman band near $\sim 296\;{cm}^{-1}$, this resonance signal provides a highly selective spectral marker for the identification of GeNCs in the composite. The Ge–Ge band is therefore well suited as a tracer for the detection and localization of marker particles in polypropylene-based materials.

The Raman maps further reveal a concentration-dependent change in nanoparticle distribution. At low loading (C01), only isolated GeNC clusters are present, indicating that the nanoparticles remain largely separated within the matrix. Increasing GeNC concentration leads first to a more homogeneous distribution (C02), followed by the formation of larger agglomerated regions at the highest loading (C03). The increasing agglomeration at higher filling levels suggests a limit to physical dispersion.

Overall, the combined spectral and spatial analyses demonstrate that Ge nanocrystals can be reliably detected within polypropylene by Raman spectroscopy. Furthermore, the method is sufficiently sensitive to distinguish between different nanoparticle concentrations and dispersion states, making it suitable for the characterization of marker-based polymer compounds. The observed trends were consistent across multiple measurements, indicating a reproducible detection of Ge nanocrystals within the polymer matrix.

## 4. Conclusions

In this study, GeNC/PP composites were successfully synthesized starting from dodecyl-functionalized GeNCs via a solvent-based intercalation (blending) approach. Using an injection molding process based on a masterbatch composite, specimens with adjustable GeNC contents were prepared. The incorporation of GeNCs within the polypropylene matrix was confirmed by Raman spectroscopy, and the corresponding spectra clearly correlate with the varying germanium contents.

Raman spectroscopy provides a non-destructive and element-specific analytical method, in contrast to alternative techniques such as X-ray fluorescence (XRF) or laser-induced breakdown spectroscopy (LIBS). Furthermore, there are no spectral overlaps as there might be in other spectra such as M/NIR. The use of a confocal Raman microscope enables measurements with high spatial resolution, allowing detailed analysis of local GeNC distributions within the polymer matrix.

This study represents a proof of concept and demonstrates that under laboratory conditions the tunable, non-toxic, surface-functionalized Ge nanocrystals can be successfully integrated into polypropylene and reliably detected due to their distinct spectroscopic signatures.

Potential limitations of the presented approach in practical situations include influences on the Raman signal arising from the polymer matrix, such as fluorescence effects, as well as the presence of common additives, pigments, stabilizers, or recycled material fractions. In addition, the observed agglomeration behavior at higher concentrations, the determination of a minimum tracer concentration required for robust detection, and effects of varying the polymer matrix as well as NC size represent important aspects that will be subjects of future research.

Further investigations into adapting the surface modification of the NCs and process optimizations in the compounding of the GeNC/PP composite, particularly with regard to very low, tracer-relevant concentrations, are necessary and should include the effect of multiple recycling cycles on tracer detectability. Future work should also focus on identifying the limits of measurement times under application-relevant conditions.

## Figures and Tables

**Table 1:** Composition and tracer concentrations of the prepared batches.

Batch	Target Concentration (wt%)	Tracer Material GeNCs (mg)	Masterbatch (mg)	Polypropylene (mg)	Total Weight (mg)
B01	-	-	-	6154.0	6154.0
C01	0.06	3.5	34.5	6117.5	6151.5
C02	0.23	14.0	136.1	6017.7	6153.7
C03	0.46	28.1	272.3	5873.4	6145.4

## Data Availability

The original contributions presented in this study are included in the article/Supplementary Materials. Further inquiries can be directed to the corresponding author.

## Supplementary Materials

The following supporting information can be downloaded at: https://www.mdpi.com/article/10.3390/ma19112185/s1, Figure S1: X-ray diffraction analysis of dodecyl-functionalized GeNCs; Figure S2: X-ray photoelectron spectroscopy of dodecyl-functionalized GeNCs; Figure S3: Raman spectroscopy of dodecyl-functionalized GeNCs; Figure S4: Raman spectroscopy mapping of GeNC/PP composites; Figure S5: Statistical assessment of Raman spectroscopy maps.

## Abbreviations

The following abbreviations are used in this manuscript:

CCD	charge coupled device
EDX	energy dispersive X-ray
FTIR	fourier transform infrared
FWHM	full width of the diffraction line at half of the maximum intensity
LIBS	laser-induced breakdown
MIR	mid infrared
NCs	nanocrystals
NIR	near-infrared
PP	polypropylene
PPWR	Packaging and Packaging Waste Regulation
PTFE	Polytetrafluorethylene
PVA	polyvinyl alcohol
PXRD	X-ray powder diffraction
TBS	tracer-based sorting
TEM	transmission electron microscopy
XPS	X-ray photoelectron spectroscopy
XRFS	X-ray fluorescence
