# Supplementary material for: Detection of Germanium Nanocrystals as Tracer Materials in Polypropylene via Raman Spectroscopy
Source: Materials (Basel). 2026 May 22;19(11):2185. doi: 10.3390/ma19112185 (PMC13257731; doi:10.3390/ma19112185)
Supplement: Supplementary file 1 [file materials-19-02185-s001.zip › materials-4305573-supplementary.pdf]

Supporting Information to

Detection of Germanium Nanocrystals as Tracer Materials in Polypropylene via Raman Spectroscopy

Content

X-ray diffraction analysis of dodecyl functionalized GeNCs 2

X-ray photoelectron spectroscopy of dodecyl functionalized GeNCs 2

Raman spectroscopy of dodecyl functionalized GeNCs 3

Raman spectroscopy mapping of GeNC/PP composites 3

Statistical assessment of Raman spectroscopy maps 4

X-ray diffraction analysis of dodecyl functionalized GeNCs

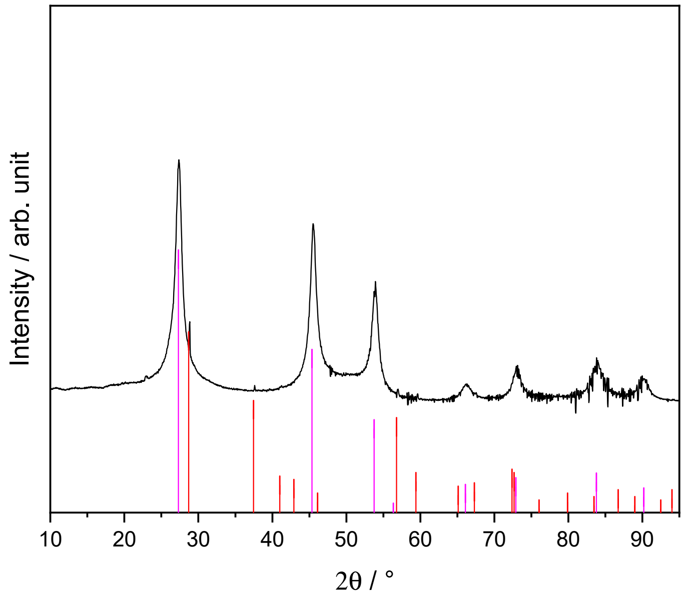

Figure S1 XRD pattern of dodecyl-functionalized GeNCs is shown. The average crystallite size that was determined using the Scherer equation is $(7.7 \pm 1.0)\ nm$. The diffraction patters of crystalline Ge (pink bars – CCD 03-065-0333) and GeO_2 (red bars – CCD 00-035-0729) references are given.

X-ray photoelectron spectroscopy of dodecyl functionalized GeNCs

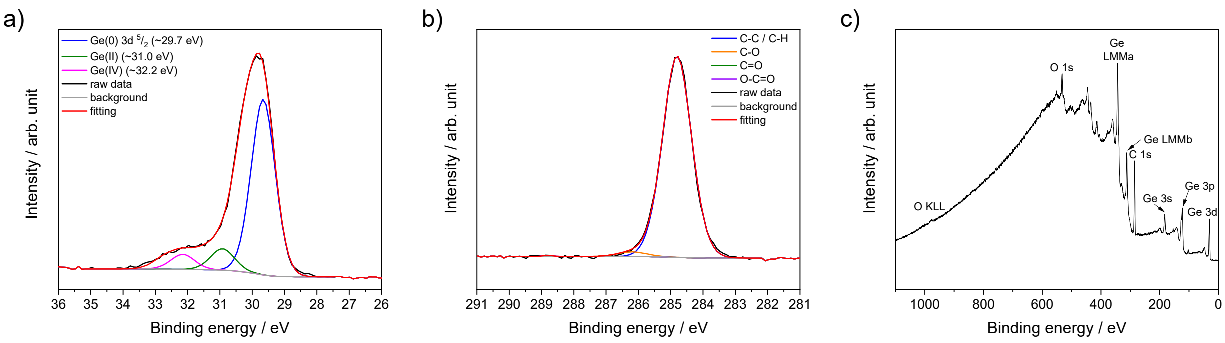

Figure S2 High resolution a) Ge 3d, b) C 1s and c) full X-ray photoelectron spectra of dodecyl-functionalized GeNCs.

Raman spectroscopy of dodecyl functionalized GeNCs

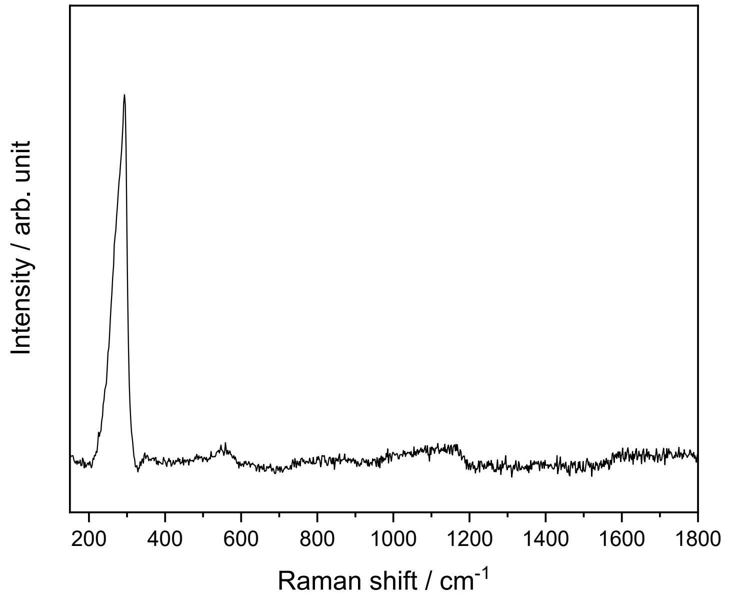

Figure S3 Raman spectrum of dodecyl-functionalized GeNCs.

Raman spectroscopy mapping of GeNC/PP composites

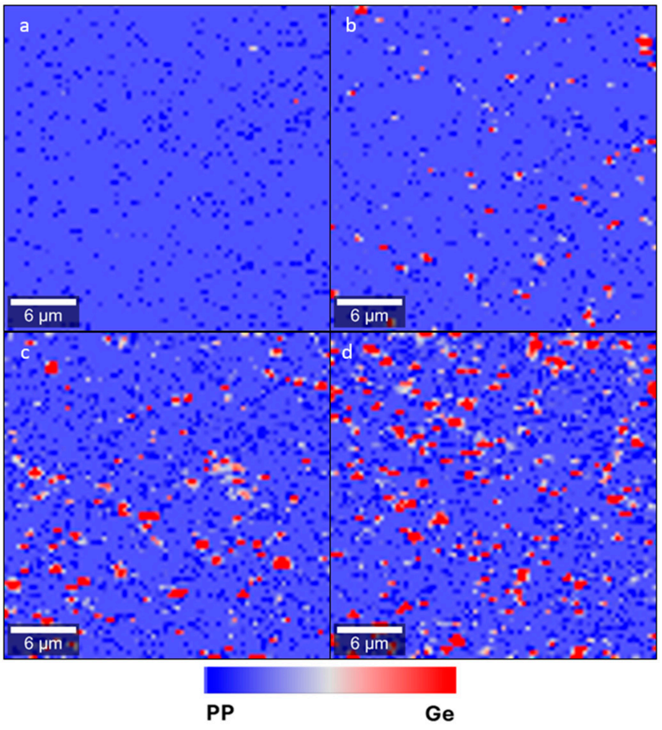

Figure S4 Two-dimensional Raman intensity maps of the $\sim 296\ cm^{-1}$ Ge–Ge phonon mode for (a) pure polypropylene (B01) and polypropylene composites with increasing germanium nanoparticle loading: (b) C01, (c) C02, and (d) C03.

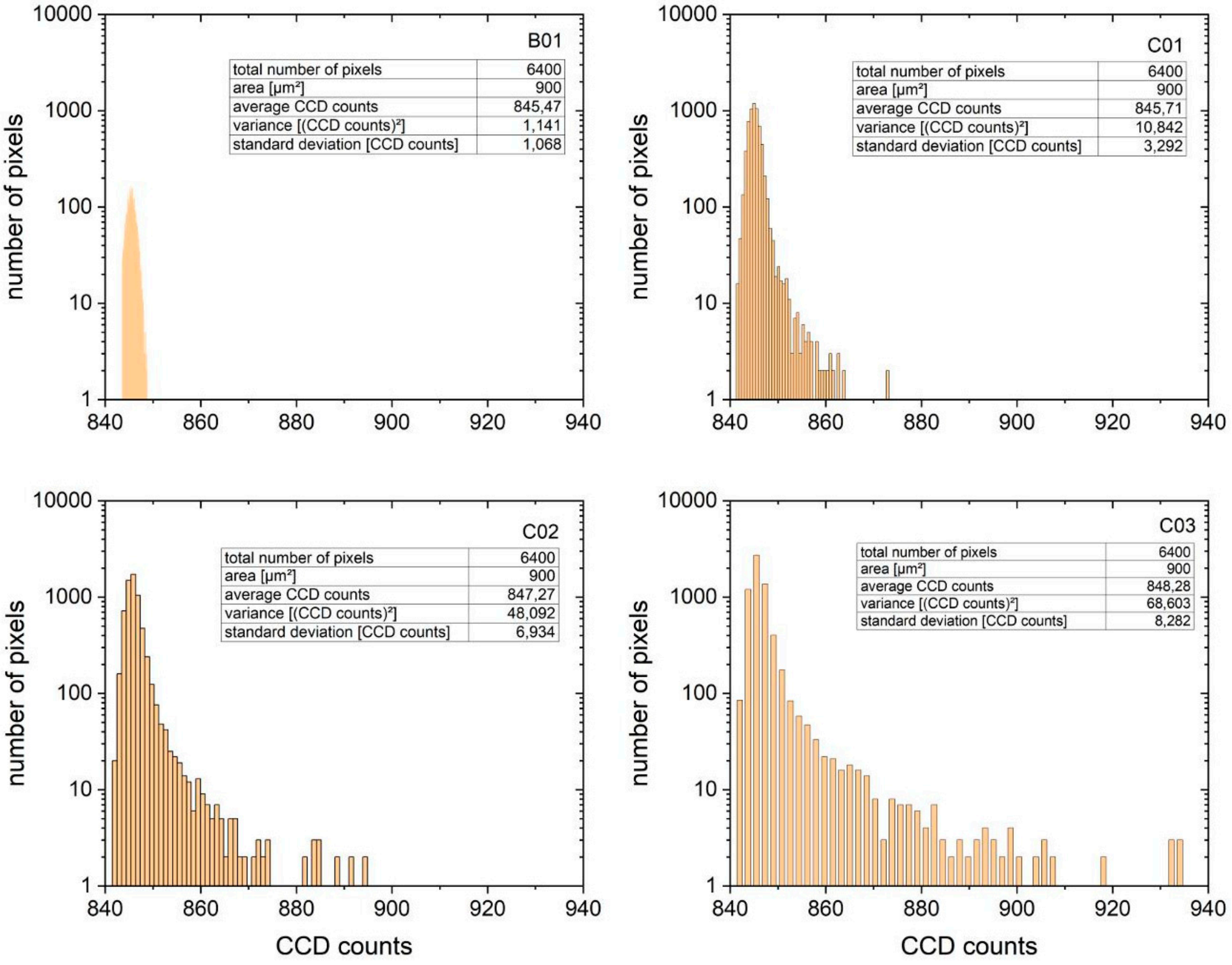

Figure S5 histograms of the Raman intensity distributions (pixel count versus signal intensity) of pure polypropylene (B01) and polypropylene composites with increasing germanium nanoparticle loading: C01, C02 and C03. The pixel intensity contributing broadens with increasing GeNC loading indicating more heterogeneity within the mapped areas.
